# Progression of Carotid Intima-Media Thickness Partly Indicates the Prevention of Hypertension among Older Individuals in the General Population

**DOI:** 10.3390/life13071588

**Published:** 2023-07-19

**Authors:** Yuji Shimizu

**Affiliations:** 1 Epidemiology Section, Division of Public Health, Osaka Institute of Public Health, Osaka 537-0025, Japan; shimizu@osaka-ganjun.jp or shimizuyuji@nagasaki-u.ac.jp; 2 Department of General Medicine, Nagasaki University Graduate School of Biomedical Sciences, Nagasaki 852-8501, Japan

**Keywords:** CIMT, CD34, platelet, hypertension, SNP, VEGF, BRAP, ALDH2, HTLV-1, angiogenesis

## Abstract

Structural atherosclerosis, as evaluated by carotid intima-media thickness (CIMT), is reported to be positively associated with hypertension. However, angiogenesis, which plays an important role in the progression of structural atherosclerosis, prevents hypertension by reducing peripheral vascular resistance. These associations evoke a contradiction: characteristics associated with the progression of structural atherosclerosis, which is related to hypertension, might prevent hypertension. To clarify novel mechanisms underlying the association between structural atherosclerosis and hypertension, multifaceted analyses are necessary. We performed several epidemiological studies based on this concept. This study summarizes those epidemiological studies and adds some discussion. Studies focusing on circulating CD34-positive cells, single-nucleotide polymorphisms (SNPs) of vascular endothelial growth factor (VEGF), SNPs in BRACA1-associated protein (BRAP), platelets, human T-cell leukemia virus type 1 (HTLV-1), and SNPs in aldehyde dehydrogenase 2 (ALDH2) have shown that active endothelial repair, which leads to the progression of structural atherosclerosis, helps prevent hypertension. These associations indicate that the progression of structural atherosclerosis could act as a marker of angiogenesis, which reduces peripheral vascular resistance. In general, a positive association between structural atherosclerosis and hypertension has been reported. However, the progression of structural atherosclerosis could act as a marker of activity that prevents hypertension via reductions in peripheral vascular resistance.

## 1. Introduction

The development of structural atherosclerosis, as evaluated based on carotid-intima media thickness (CIMT), is an established cardiovascular risk factor [1,2]. Increased CIMT indicates a process of endothelial repair.

Aging is a process that increases the need for endothelial repair but reduces endothelial repair activity. Thus, the absence of CIMT development does not always indicate a healthy endothelium, which is why no significant associations between yearly progression of CIMT and cardiovascular disease have been observed [3].

Angiogenesis is also necessary for structural atherosclerosis to develop [4]. The inhibition of angiogenesis induces hypertension by increasing the peripheral vascular resistance [5].

The development of structural atherosclerosis could indicate angiogenesis, which contributes to lower peripheral blood pressure. Since angiogenesis reduces peripheral blood pressure, the development of structural atherosclerosis could have a beneficial influence on hypertension prevention.

However, a significant positive association between structural atherosclerosis and hypertension has been reported [6]. Therefore, the positive association between hypertension and structural atherosclerosis seems to be paradoxical. The progression of structural atherosclerosis, which is positively associated with hypertension, also plays an important role in hypertension prevention.

Oxidative stress, which induces both hypertension [7] and structural atherosclerosis [8], increases with aging. Since the development of structural atherosclerosis and hypertension are biological reactions to hypoxia and oxidative stress, the development of atherosclerosis and hypertension should have a beneficial influence on the ability to perform activities of daily living.

Based on this concept, we have conducted several epidemiological studies that focused on circulating CD34-positive cell count, single-nucleotide polymorphisms (SNPs) of vascular endothelial growth factor (VEGF), SNPs in BRACA1-associated protein (BRAP), platelet count, human T-cell leukemia virus type 1 (HTLV-1), and SNPs in aldehyde dehydrogenase 2 (ALDH2).

Here, we summarize our previous epidemiological studies and add some discussion about a novel mechanism underlying endothelial maintenance.

## 2. Materials and Methods

### 2.1. Studies

To clarify a potential novel mechanism underlying endothelial repair, we conducted several studies which provided us with multiple perspectives.

First, we focus on the cell type that directly contributes to endothelial repair. In conjunction with platelets, CD34-positive cells contribute to the crucially important process of endothelial repair by differentiating into endothelial cells [9], megakaryocytes [10], foam cells, and macrophages [11]. Therefore, to investigate the role of circulating CD34-positive cells in vascular remodeling, we have performed a circulating CD34-positive cell-related survey. This survey was conducted as an addition to the annual health check-up recommended by the Japanese government. The details of the survey have been described elsewhere [12].

Second, because angiogenesis contributes to the development of structural atherosclerosis [4], we focused on a VEGF, a genetic factor that contributes to angiogenesis. [13]. Since VEGF polymorphism rs3025039 is reported to be inversely associated with serum VEGF levels in healthy individuals [14], we also conducted epidemiological studies with data on rs3025039 SNPs. The details of the survey have been described elsewhere [15].

Third, the majority of HTLV-1 carriers remain asymptomatic throughout their lives [16]. HTLV-1 infection enhances inflammation by promoting the NF-κB pathway [17]. Since the activation of the NF-κB pathway promotes the production of platelet activation proteins [18] and platelets play an important role in endothelial repair [9,10,11], HTLV-1 infection might also influence the mechanism underlying endothelial repair. Several studies using data on HTLV-1 infection were also conducted. The details of our surveys about HTLV-1 have been described elsewhere [19].

In addition, ethanol exposure dramatically inhibits NF-κB [20] and directly attenuates platelet activation [21]. Therefore, avoiding ethanol exposure might have the beneficial effect of activating endothelial repair. ALDH2 is a key enzyme in alcohol metabolism which relates to alcohol tolerance. Since ALDH2 gene polymorphisms are widely present in East Asians [22,23], including Japanese individuals, ALDH2 might also contribute to endothelial repair partly by avoiding ethanol exposure. We have also conducted a survey with data on SNPs related to ALDH2 [23].

Participants of those epidemiological studies participated in an annual health check-up in the city of Goto and the town of Saza. Both are located in Nagasaki Prefecture, in western Japan. The city of Goto is located on a remote island. We evaluated cardio–ankle vascular index (CAVI), SNPs, and HTLV-1 infection only in the city of Goto. The town of Saza is a bedroom community adjacent to the city of Sasebo. CIMT was evaluated at both study locations.

These studies were approved by the ethics committee of the Nagasaki University Graduate School of Biomedical Sciences (project registration numbers, 14051404-1 to 14051404-13). Written consent forms were made available to ensure that the participants understood the objectives of the studies. Informed consent was obtained from all participants. All procedures performed in this study were in accordance with the ethical standards of the institutional research committee and the 1964 Declaration of Helsinki and its later amendments.

### 2.2. Data Collection

#### 2.2.1. General Measurements

Each participant’s medical history was ascertained by a specially trained interviewer. An automatic body composition analyzer (BF-220; Tanita, Tokyo, Japan) was used to calculate body mass index (BMI, kg/m^2^) after measuring height and weight. After at least 5 min of rest, blood pressure was measured in the sitting position using a blood pressure device (HEM-907; Omron, Kyoto, Japan). Fasting blood samples were collected in an EDTA-2K tube and a siliconized tube. High-density lipoprotein cholesterol (HDLc), low-density lipoprotein cholesterol (LDLc), triglycerides, glycohemoglobin (HbA1c), aspartate transaminase (AST), alanine aminotransferase (ALT), gamma-glutamyl transpeptidase (γ-GTP), and serum creatinine were measured using standard procedures at SRL, Inc. (Tokyo, Japan). As a marker of renal function, estimated glomerular filtration rate (eGFR) was calculated with an established method adapted by a working group of the Japanese Chronic Kidney Disease Initiative: eGFR (mL/min/1.73 m^2^) = 194 × (serum creatinine (enzyme method))^−1.094^ × (age)^−0.287^ × (0.739 for women) [24].

#### 2.2.2. Carotid Intima-Media Thickness (CIMT)

CIMT in the left and right common carotid arteries was measured using a LOGIQ Book XP with a 10 MHz transducer (GE Healthcare, Milwaukee, WI, USA). Semi-automated digital edge-detection software (Intimascope; MediaCross, Tokyo, Japan) [25] was used to calculate mean and maximum left and right common CIMT values. This software semi-automatically recognizes the edges of the internal and external membranes of the artery and automatically determines distances at a sub-pixel level, estimated to be 0.01 mm [26]. In our epidemiological studies, structural atherosclerosis was defined as CIMT ≥ 1.1 mm.

#### 2.2.3. Cardio–Ankle Vascular Index (CAVI)

Brachial–ankle pulse wave velocity (PWV) measurements are generally used to evaluate functional arterial stiffness. Since PWV measurements can be strongly affected by blood pressure [27], the cardio–ankle vascular index (CAVI) was recently developed in Japan to avoid the confounding effects of blood pressure [28]. In the current studies, the CAVI was determined using a VaSera VS-1000 vascular screening system (Fukuda Denshi, Tokyo, Japan) with the participant resting in a supine position.

#### 2.2.4. Measurement of Circulating CD34-Positive Cell Count

To measure CD34-positive cell count, blood samples were collected in heparin sodium tubes. CD34-positive cells were measured in blood samples from the heparin sodium tube within 24 h of sample collection using BD Trucount^TM^ technology (Beckton Dickinson Biosciences, San Jose, CA, USA), an accurate and reproducible single-platform assay cited in the International Society of Hematotherapy and Graft Engineering (ISHAGE) guidelines [29] and supported by automated software in the BD FACSCanto II system. Approximately 30 min is required to measure the circulating CD34-positive cell count for each sample. Measurement of circulating CD34-positive cell count requires a fresh sample, within 24 h of blood collection. Therefore, CD34-positive cell count can be measured in a maximum of 20 samples each day. Thus, we limited the measurement of CD34-positive cell count to men aged 60–69 years who participated in an annual health check-up.

#### 2.2.5. Genotyping of the Single-Nucleotide Polymorphisms (SNPs)

Genomic DNA was extracted from 2 mL of peripheral whole blood using Gene Prep Star NA-480 (Kurabo Industries Ltd., Osaka, Japan). Genotyping of the SNPs rs3025039 (VEGF), rs3025020 (VEGF), rs3782886 (BRAP), and rs671 (ALDH2) was conducted using TaqMan assays and a LightCycler 480 thermal cycling platform (Roche Diagnostics, Basel, Switzerland).

#### 2.2.6. Detection of Human T-Cell Leukemia Virus 1 (HTLV-1) Infection

A chemiluminescent enzyme immunoassay (CLEIA) kit (Fujirebio Inc., Tokyo, Japan) was used to detect HTLV-1 infection at SRL, Inc. Confirmatory tests for HTLV-1 were not performed in this study. Therefore, detection of serum anti-HTLV-1 antibodies was performed, but not detection of HTLV-1 viral DNA. However, we believe that the lack of confirmatory testing for HTLV-1 infection had a limited effect. Our previous study that involved real-time reverse transcription polymerase chain reaction with a hydrolysis probe and Western blotting showed a low false-positive rate (1.2%) [30].

## 3. Results

To clarify a novel mechanism underlying endothelial repair, several epidemiological studies based on data from the city of Goto and the town of Saza were performed. Parts of those studies are described below.

### 3.1. CIMT and CAVI in Relation to Circulating CD34-Positive Cell Count (Figure 1)

**Figure 1:**
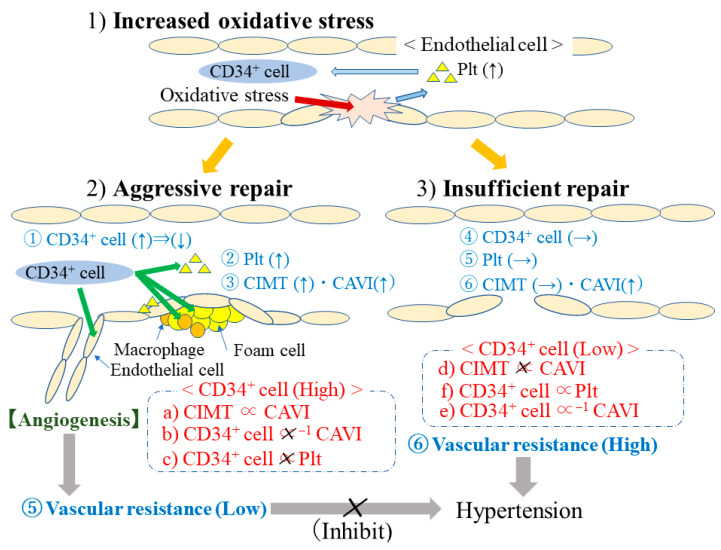
Mechanism of endothelial repair in relation to CD34^+^ cell. CD34^+^ cell: CD34-positive cell. CAVI: Cardio–Ankle Vascular Index. CIMT: Carotid Intima-Media Thickness. Plt: Platelet.

A cross-sectional study with 249 men aged 60–69 years [31] revealed a significant positive association between CIMT and CAVI only in individuals with high circulating CD34-positive cell counts (Figure 1a,d). The multivariable standardized parameter estimate [β] was 0.22 (*p* = 0.028) for individuals with high circulating CD34-positive cell counts (median≤) and −0.02 (*p* = 0.865) for individuals with low circulating CD34-positieve cell counts (<median). The study also revealed that logarithmic values of circulating CD34-positive cell count are inversely associated with CAVI among those with low circulating CD34-positive cell counts (β = −0.22, *p* = 0.014) but not among those with high ones (β = −0.04, *p* = 0.638), (Figure 1e,b). In this study, platelet count was significantly positively associated with circulating CD34-positive cell count only in those with low circulating CD34-positive cell counts. The simple correlation coefficient (r) and *p*-value between platelet count and circulating CD34-positive cell count in individuals with low and high circulating CD34-positive cell counts were r = 0.23, *p* = 0.009 and r = −0.02, *p* = 0.848, respectively (Figure 1f,c).

### 3.2. Gamma-Glutamyl Transpeptidase (γ-GTP), Structural Atherosclerosis, and Hypertension in Relation to Circulating CD34-Positive Cell Count

A cross-sectional study with 562 men aged 60–69 years [6] revealed a significant positive association between hypertension and structural atherosclerosis; the multivariable odds ratio (OR) (95% confidence interval [CI]) with adjustment for known cardiovascular risk factors (age, BMI, alcohol consumption, smoking status, systolic blood pressure, HDLc, triglycerides, and HbA1c) was 2.09 (1.30, 3.35).

For individuals with high CD34-positive cell counts (≤median), γ-GTP was significantly and positively associated with structural atherosclerosis (OR for the log-transformed value of γ-GTP = 2.26 (1.32, 3.86)) but not with hypertension (OR = 0.77 (0.51, 1.17)).

Among those with low CD34-positive cell counts, γ-GTP was not significantly associated with structural atherosclerosis (OR = 0.92 (0.51, 1.68)) but was significantly and positively associated with hypertension (OR = 1.99 (1.27, 3.12)).

### 3.3. Vascular Endothelial Growth Factor (VEGF) Polymorphisms and Structural Atherosclerosis among Hypertensive Elderly Individuals

VEGF contributes to the progression of angiogenesis [13]. In a cross-sectional study with 1793 older hypertensive Japanese individuals aged 60–89 years [15], the minor allele of polymorphism rs3025039, which was reported to be inversely associated with the serum concentration of VEGF [14], was inversely associated with structural atherosclerosis, independent of known confounders. Since the minor allele of polymorphism rs3025020 was positively associated with the serum concentration of VEGF [14], the fully adjusted model for this analysis was adjusted for sex, age, rs3025020 genotype, BMI, drinking status, smoking status, HDLc, triglycerides, and HbA1c. The fully adjusted OR (95% CI) for structural atherosclerosis with the minor allele of rs3025039 was 0.78 (0.64, 0.96).

### 3.4. Platelets, Circulating CD34-Positive Cells, and CIMT by Hypertension Status (Figure 2)

A cross-sectional study with 567 men aged 60–69 years indicated that platelet count is an indicator of endothelial repair activity and that the presence of hypertension might mask the beneficial effects of circulating CD34-positive cells [32].

**Figure 2:**
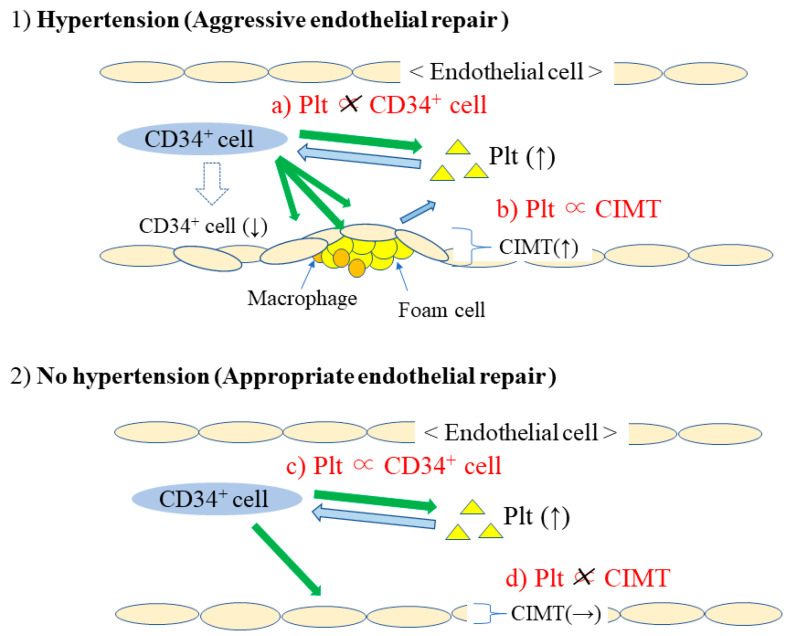
Mechanism of endothelial repair in relation to platelet count. CD34^+^ cell: CD34-positive cell. CAVI: Cardio–Ankle Vascular Index. CIMT: Carotid Intima-Media Thickness. Plt: Platelet.

In individuals without hypertension, platelet count was not significantly correlated with CIMT (β = −0.05, *p* = 0.356) (Figure 2d), but platelet count was significantly positively correlated with the natural log of circulating CD34-positive cell count (β = 0.26, *p* < 0.001) (Figure 2c).

In individuals with hypertension, a significant positive correlation was seen between platelet count and CIMT (β = 0.19, *p* = 0.008) (Figure 2b), whereas no significant correlation was seen between platelet count and the natural log of circulating CD34-positive count (β = 0.11, *p* = 0.119) (Figure 2a).

### 3.5. Platelets and Hypertension by Levels of Circulating CD34-Positive Cell Count

To clarify the association between platelet count and hypertension in relation to levels of circulating CD34-positive cell count, a cross-sectional study with 580 Japanese men aged 60–69 years was conducted [33]. Platelet count was positively associated with hypertension among participants with a low CD34-positive cell count. After adjustment for known cardiovascular risk factors (age, BMI, alcohol consumption, smoking status, HDLc, HbA1c, triglycerides, AST, and estimated glomerular filtration rate (eGFR), lipid lowering medication use, and glucose lowering medication use), the OR and 95% CI for hypertension with each 1 standard deviation (SD) increment in platelet count (5.24 × 10^4^/μL), was 1.47 (1.12, 1.91) among participants with a low CD34-positive cell count and 0.91 (0.71, 1.18) among those with a high CD34-positive cell count.

### 3.6. HTLV-1, Structural Atherosclerosis, and Hypertension (Figure 3)

**Figure 3:**
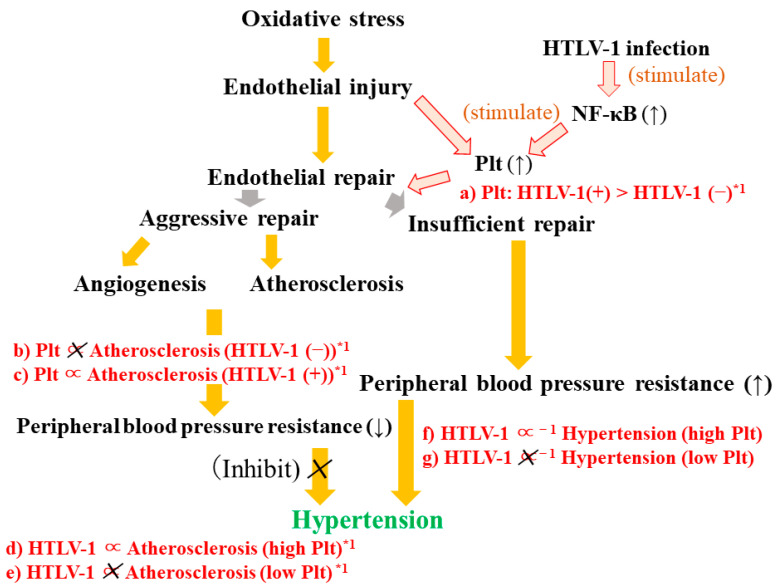
Mechanism of endothelial repair in relation to HTLV-1 infection. Atherosclerosis: Structural atherosclerosis. Plt: Platelet. *1: Indicates the association observed among non-hypertensives. Associations shown in red (a~e).

A cross-sectional study with 2989 Japanese individuals aged 60–99 years [19] found HTLV-1 infection to be significantly inversely associated with hypertension only in individuals with high platelet counts (≥second tertile) (Figure 3f,g). The fully adjusted (adjusted for sex, age, BMI, HDLc, triglycerides, HbA1c, white blood cell count, and γ-GTP) ORs and 95% CIs were 0.75 (0.62, 0.92) overall, 0.64 (0.50, 0.82) for high platelet counts, and 1.01 (0.72, 1.42) for low platelet counts (first tertile).

Among non-hypertensive individuals, platelet count was significantly positively associated with structural atherosclerosis among those with HTLV-1 infection but not among those without HTLV-1 infection (Figure 3c,b). The ORs (95% CIs) for structural atherosclerosis with each 1 SD increment in platelet count were 1.44 (1.05, 1.97) for those with HTLV-1 infection and 1.06 (0.88, 1.28) for those without HTLV-1 infection.

Furthermore, among non-hypertensive individuals, HTLV-1 infection was significantly positively associated with structural atherosclerosis in individuals in the highest platelet count tertile (2.11 [1.15, 3.86]), but not in those with low platelet count (first or second platelet count tertiles) (0.89 [0.57, 1.39]) (Figure 3d,e).

In addition, among non-hypertensive individuals, those with HTLV-1 infection had significantly higher platelet counts (*p* = 0.006) than those without HTLV-1 infection (Figure 3a). The sex- and age-adjusted values (least mean square ± standard error (SE)) for platelet count were 23.1 ± 0.4 (×10^4^/μL) for those with HTLV-1 infection and 21.8 ± 0.2 (×10^4^/μL) for those without HTLV-1 infection.

### 3.7. Reticulocytes, Hypertension, and Structural Atherosclerosis (Figure 4)

**Figure 4:**
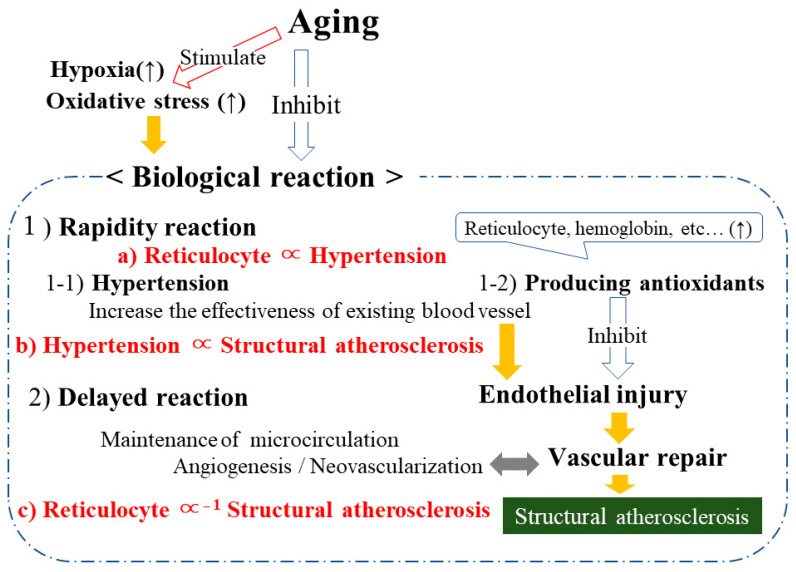
Potential biological reaction for hypoxia and oxidative stress.

A cross-sectional study with 2098 elderly individuals aged 60–89 years revealed that reticulocyte count is significantly positively associated with hypertension and inversely associated with structural atherosclerosis [34]. In a fully adjusted model for the analysis between reticulocytes and hypertension, sex, age, BMI, HDLc, triglycerides, HbA1c, γ-GTP, white blood cell count, and eGFR were regarded as confounders. Systolic blood pressure was further adjusted for the analysis between reticulocytes and atherosclerosis. After adjusting for known confounders, the ORs (95% CIs) for hypertension and atherosclerosis with each 1 SD increment in reticulocyte count were 1.12 (1.01, 1.25) and 0.83 (0.72, 0.94), respectively (Figure 4a,c). This study also showed a significant positive association between hypertension and structural atherosclerosis. The fully adjusted (adjusted for sex, age, BMI, HDLc, triglycerides, HbA1c, γ-GTP, white blood cell count, and eGFR) OR (95% CI) for structural atherosclerosis and hypertension was 1.34 (1.03, 1.74) (Figure 4b).

### 3.8. Hemoglobin and Hypertension

A cross-sectional study with 3203 individuals aged 30–79 years without anemia showed a positive association between hemoglobin levels and hypertension. For each 1 SD increment in hemoglobin, the OR (95% CI) for hypertension was 1.21 (1.05, 1.40) for men and 1.25 (1.13, 1.39) for women after adjusting for known cardiovascular risk factors (age, BMI, smoking status, drinking status, history of cardiovascular disease, diabetes, triglycerides, ALT, γ-GTP, and eGFR) [35].

### 3.9. Hemoglobin and Hypertension by Platelet Count (Figure 5)

**Figure 5:**
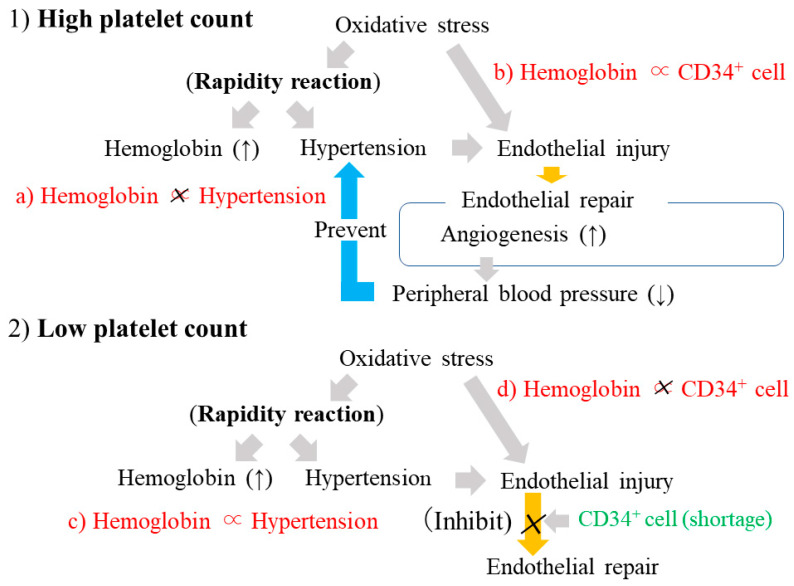
Association between hemoglobin and hypertension by platelet levels. CD34^+^ cell: CD34-positive cell.

A cross-sectional study with 222 Japanese men aged 60–69 years was conducted to clarify the influence of platelets on the association between hemoglobin and hypertension [36]. The positive association between hemoglobin levels and hypertension was limited to individuals with lower platelet count (at or under the median value) (Figure 5a,c). The adjusted OR and 95% CI for hypertension with each 1 SD increment in hemoglobin (1.0 g/dL) was 2.09 (1.26, 3.48) for those with a lower platelet count and 1.07 (0.68, 1.67) for those with a higher platelet count. Among men with a lower platelet count, no significant correlations between hemoglobin and circulating CD34-positive cell count were observed (β = −0.06, *p* = 0.603) (Figure 5d), but a significant positive correlation was observed for participants with a higher platelet count (β = 0.29, *p* = 0.004) (Figure 5b).

### 3.10. BRAP rs3782886 and Platelet Count in Relation to Hypertension (Figure 6)

To clarify the association between SNP rs3782886 in BRAP and hypertension-related high platelet count, a cross-sectional study with 988 Japanese individuals aged 60–89 years was conducted [37]. 

**Figure 6:**
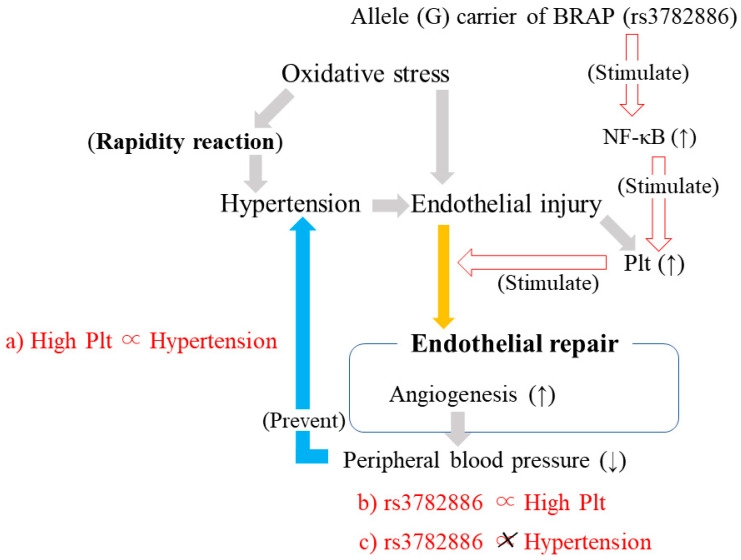
Association among high platelet count, hypertension, and BRAP (rs3782886). Plt: Platelet.

High platelet count was defined as being the highest platelet count tertile. High platelet count was found to be independently positively associated with hypertension (Figure 6a), while rs3782886 was independently associated with high platelet count (Figure 6b). After adjusting for classical cardiovascular risk factors, the OR and 95% CI for high platelet count and hypertension was 1.34 (1.02, 1.77). With the non-minor homo (A/A, A/G) of rs3782886 as the reference group, the adjusted OR and 95% CI for high platelet count and minor homo (G/G) was 2.40 (1.30, 4.42).

This study also revealed no significant associations between BRAP rs3782886 and hypertension (Figure 6c). With non-minor homo as the reference group, the adjusted OR and 95% CI for hypertension and minor homo was 1.08 (0.60, 1.98).

### 3.11. Potential Mechanism Underlying the Association between SNP (BRAP and ALDH2) and Hypertension (Figure 7)

**Figure 7:**
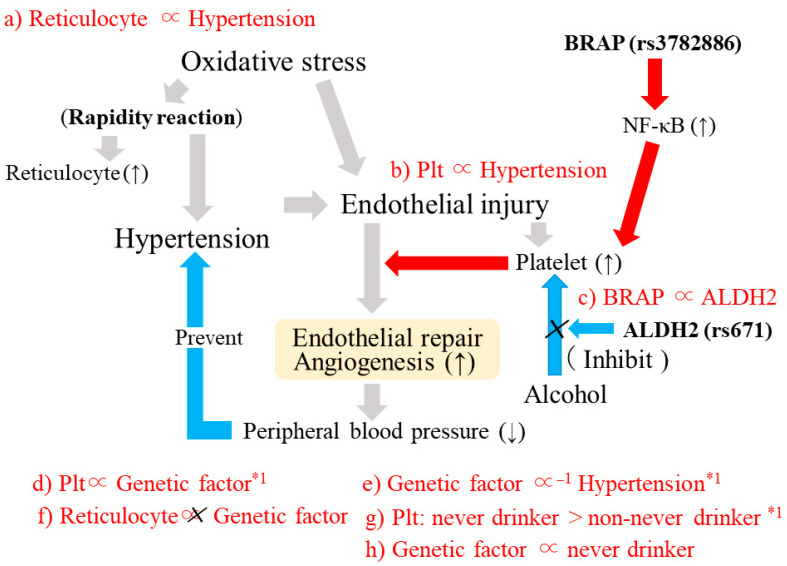
BRAP (rs3782886), ALDH2 (rs671), and hypertension. *1: Observed only among participants with high reticulocyte counts. Plt: Platelet. Red arrows indicate stimulate. Light blue allows indicate inhibit.

A multifaceted analysis was performed with a simple model of elderly individuals in the general population that included 1313 Japanese individuals aged 60–98 years [23]. Both platelet count and reticulocyte count were revealed to be positively associated with hypertension; after adjusting for known cardiovascular risk factors, the ORs (95% CIs) for hypertension and 1 SD increments in platelet count and reticulocyte count were 1.22 (1.08, 1.38) and 1.19 (1.03, 1.36), respectively (Figure 7a,b).

Participants were stratified by the median reticulocyte count. Strong linkage disequilibrium (LD) values have been reported between rs3782886 and rs671 [38]. The simple correlation coefficient (r) between the number of minor alleles of rs671 and rs3782886 was 0.94 (*p* < 0.001) for men and women (Figure 7c).

Although no significant correlations between the number of minor alleles of rs3782886 and platelet count were observed among participants with low reticulocyte counts (*p* = 0.480), platelet count was significantly positively correlated with minor allele of rs3782886 among those with high reticulocyte counts (*p* = 0.005) (Figure 7d). Regarding reticulocyte count, for both individuals with high (*p* = 0.407) and low reticulocyte counts (*p* = 0.790), no significant correlations were observed between the minor allele of rs3782886 and reticulocyte count (Figure 7f).

For both rs3782886 and rs671, the number of minor alleles was significantly positively associated with never drinker status; the age-adjusted OR (95% CI) for never drinker status and the number of minor alleles of rs3782886 was 5.20 (3.63, 7.45) for men and 3.01 (2.06, 4.39) for women. For rs671, the corresponding values were 5.47 (3.78, 7.92) for men and 3.49 (2.32, 5.25) for women (Figure 7h).

Platelet count was significantly higher in never drinkers than in non-never drinkers only among participants with high reticulocyte counts (Figure 7g). The sex-adjusted platelet count (mean ± standard error (8 × 10^4^/μL)) was 23.1 ± 0.3 for never drinkers (*n* = 369) and 22.2 ± 0.3 for non-never drinkers (*n* = 288) (*p* = 0.046). Among the participants with low reticulocyte counts, no significant associations were observed between sex-adjusted platelet count and drinking status (*p* = 0.444). The sex-adjusted platelet count was 22.7 ± 0.3 for never drinkers (*n* = 425) and 22.3 ± 0.4 for non-never drinkers (*n* = 231).

Independent of known cardiovascular risk factors, the minor alleles of rs3782886 and rs671 were significantly inversely associated with hypertension in participants with high reticulocytes counts. The fully adjusted OR (95% CI) was 0.72 (0.55, 0.96) for rs3782886 and 0.72 (0.54, 0.96) for rs6719. No associations were observed in individuals with low reticulocyte counts. The corresponding values were 1.05 (0.79, 1.39) and 1.08 (0.81, 1.45), respectively (Figure 7e).

### 3.12. Structural Atherosclerosis, Functional Atherosclerosis, and LDL Cholesterol (LDLc)

To clarify the association between LDLc and atherosclerosis (structural and functional) among older individuals, a cross-sectional study with 1458 Japanese individuals aged 60–79 years was conducted. In this study, LDLc was significantly positively associated with structural atherosclerosis but significantly inversely associated with functional atherosclerosis [39]. For each 1 SD increment in LDLc, the OR (95% CI) was 1.28 (1.10, 1.50) for structural atherosclerosis and 0.85 (0.75, 0.96) for functional atherosclerosis after adjusting for known cardiovascular risk factors (sex, age, BMI status, hypertension, drinking status, smoking status, low HDLc, diabetes, high triglycerides, and lipid lowering medication use).

## 4. Discussion

As shown in Figure 1, aggressive endothelial repair results in structural and functional atherosclerosis, while insufficient endothelial repair results in functional atherosclerosis but not structural atherosclerosis (Figure 1➂,➅).

Platelets, which are complex anucleate cells, contribute to vascular homeostasis [40]. Upon vascular injury, platelets become activated and contribute to both rapid adhesion to the exposed subendothelial matrix [41] and induce proliferation of CD34-positive cells [11]. Platelets also induce CD34-positive cell differentiation into megakaryocytes [10], endothelial cells [9], macrophages, and foam cells [11].

Megakaryocytes are a known source of platelets [10]. To increase endothelial repair, the number of CD34-positive cells as well as the number of endothelial cells, macrophages, foam cells, and platelets should be increased (Figure 1➀,➁). However, during aggressive endothelial repair, many CD34-positive cells differentiate into mature cells (CD34-negative cells) (Figure 1➀). Since increases in platelet count induce decreases in CD34-positive cell count by inducing their differentiation into megakaryocytes [10], aggressive endothelial repair disturbs the positive correlation between platelet and circulating CD34-positive cell counts shown in our study [31] (Figure 1➀,➁,➃,➄) (Figure 1c,f).

The development of pathological atherosclerosis, which is related to increased CIMT, requires sufficient numbers of macrophages [42] and foam cells [43]. Therefore, sufficient numbers of CD34-positive cells are mandatory for the development of structural atherosclerosis, as evaluated based on CIMT increase [44] (Figure 1➂). In addition, because shortages of CD34-positive cells lead to the progression of functional atherosclerosis, circulating CD34-positive cell count was inversely associated with CAVI only among participants with low circulating CD34-positive cell counts (Figure 1b,e).

### 4.1. Aggressive Endothelial Repair and Insufficient Endothelial Repair

During aging, there is more oxidative stress [45,46]. Oxidative stress is a major factor that disturbs vascular health among older individuals because both hypertension [7] and endothelial dysfunction [8] are induced by oxidative stress. 

However, aging is also associated with declining hematopoietic activity that results in reducing CD34-positive cell production [47,48]. Therefore, aging is a process that increases the need for endothelial repair and decreases endothelial repair activity. Thus, the risk for insufficient endothelial repair is higher for older individuals than younger individuals. Circulating CD34-positive cell count acts as a marker of endothelial repair activity [49].

In our study, a significant positive association between CIMT and CAVI was only observed in participants with high circulating CD34-positve cell counts. This study also revealed a significant inverse association between circulating CD34-positive cell count and CAVI only in participants with low circulating CD34-positve cell counts [31]. Therefore, individuals with sufficient numbers of circulating CD34-positive cells have aggressive endothelial repair that leads to both structural atherosclerosis and functional atherosclerosis (Figure 1➂). A shortage of CD34-positive cells, which results in insufficient endothelial repair, leads to the development of functional atherosclerosis but not structural atherosclerosis (Figure 1➅). Since aggressive endothelial repair disturbs the positive correlation between platelet and circulating CD34-positive cell counts via the consumption of CD34-positive cells (Figure 1➀), the positive association between platelet and circulating CD34-positive cell counts was only observed in those with low circulating CD34-positve cell counts in our study [31] (Figure 1c,f).

Since CD34-positive cells also contribute to the maintenance of the microcirculation by promoting angiogenesis [50] and neovascularization [51], individuals with aggressive endothelial repair also have lower peripheral blood pressure resistance, which has the beneficial effect of preventing hypertension [5,6].

### 4.2. Circulating CD34-Positive Cell Counts and the Beneficial Effect of Preventing Hypertension, Which Is Related to the Development of Structural Atherosclerosis

Serum γ-GTP could act as a marker of oxidative stress [52,53]. In a cross-sectional study, among those with enough circulating CD34-positive cells, γ-GTP levels were revealed to be positively associated with structural atherosclerosis but not with hypertension. Among those with a shortage of circulating CD34-positive cells, no significant associations between γ-GTP levels and structural atherosclerosis were observed, but a significant positive association between γ-GTP levels and hypertension was observed [6]. Those findings indicate that aggressive endothelial repair, which leads to the development of structural atherosclerosis, has the beneficial effect of preventing hypertension.

Since angiogenesis is necessary for structural atherosclerosis to develop [4], the development of structural atherosclerosis could act as a marker of the progression of angiogenesis, which reduces peripheral blood pressure (Figure 1➄,➅). Since a significant positive association between hypertension and structural atherosclerosis has been observed [6], oxidative stress itself induces both hypertension and structural atherosclerosis.

### 4.3. VEGF Polymorphisms and Structural Atherosclerosis among Elderly Individuals with Hypertension

Increased levels of oxidative stress induce hypertension [7] and structural atherosclerosis [8]. Since angiogenesis is necessary for the development of structural atherosclerosis [4], individuals who have a genetic disadvantage in the progression of angiogenesis might have a disadvantage in the development of structural atherosclerosis. Next, a study was performed among participants with hypertension. In our cross-sectional study, the minor allele of VEGF polymorphism rs3025039 was inversely associated with structural atherosclerosis among elderly individuals with hypertension [15]. Since the inhibition of angiogenesis induces hypertension [5], this study indicates that individuals who have a genetic disadvantage in regard to the development of angiogenesis also might have a disadvantage in terms of developing structural atherosclerosis (Figure 1➄,➅).

### 4.4. Platelet Count as a Marker of Endothelial Repair Activity

In conjunction with CD34-positive cells, platelets contribute to endothelial repair. Activated platelets induce the proliferation of CD34-positive cells [11]. Platelets also induce CD34-positive cell differentiation into megakaryocytes [10], endothelial cells [9], macrophages, and foam cells [11].

Hypertension is the most common factor that injures the endothelium. Among the general population, an analysis limited to individuals with hypertension could more clearly show the influence of aggressive endothelial repair. An analysis limited to individuals without hypertension could more clearly show the influence of appropriate endothelial repair. According to this concept, analyses stratified by hypertension status were performed to focus on the role of platelets in endothelial repair [32]. No significant correlations between platelet count and circulating CD34-positive cell count were observed among hypertensive participants (Figure 2a). However, in non-hypertensive participants, platelet count was significantly positively associated with circulating CD34-positive cell count (Figure 2c). Since aggressive endothelial repair reduces the circulating CD34-positive cell count, those associations indicate that analyses among hypertensive participants could be influenced by the aggressive endothelial repair. This study also found that platelet count is positively associated with CIMT only in participants with hypertension (Figure 2b,d). Therefore, platelet count could indicate endothelial repair activity. In hypertensive individuals, platelet count indicates increasing CIMT, whereas platelet count indicates the ability to produce CD34-positive cells for non-hypertensive individuals.

The positive correlation between circulating CD34-positive cell count and platelet count was observed among individuals with insufficient endothelial repair, which is related to a shortage of circulating CD34-positive cells [31] (Figure 1). This positive correlation was also observed for non-hypertensive individuals but not for hypertensive individuals [32]. Therefore, the correlation between platelet count and circulating CD34-positive cell count could be an efficient tool for evaluating endothelial repair activity in a research setting. Among those with aggressive endothelial repair, no significant correlations between platelet count and circulating CD34-positive cell count were observed. However, among those with insufficient or appropriate levels of endothelial repair, a significant positive correlation between platelet count and circulating CD34-positive cell count could be observed [31,32].

### 4.5. Platelets and Hypertension by Circulating CD34-Positive Cell Count

In conjunction with platelets, CD34-positive cells [9,10,11] contribute to endothelial repair. Aggressive endothelial repair could induce a reduction in circulating CD34-positive cell counts due to a consumption that is much stronger than the effect on platelet count.

Therefore, among participants with low circulating CD34-positive cell counts, platelet count could indicate insufficient endothelial repair related to hypertension. In a cross-sectional study, platelet count was significantly positively associated with hypertension among those with low (<median) circulating CD34-positive cell counts but not among those with high circulating CD34-positive cell counts (≥median) [33]. Thus, inappropriate endothelial repair could be a risk factor for hypertension.

### 4.6. Human T-Cell Leukemia Virus Type 1 (HTLV-1), Structural Atherosclerosis, and Hypertension

HTLV-1, a human retrovirus, has been shown to induce adult T-cell leukemia/lymphoma [54], myelopathy/tropical spastic paraparesis, sensorimotor polyneuropathy [55], and optic neuritis. However, the majority of carriers remain asymptomatic throughout their lives [16,56,57].

HTLV-1 induces inflammation via the p40Tax transactivator [58,59], possibly by activating the NF-κB pathway [17]. The activation of the NF-κB pathway could promote the production of platelet activation proteins [18]. Thus, HTLV-1 carriers have significantly more platelets than non-carriers (Figure 3a).

Since platelet count could indicate endothelial repair activity [32], including the development of angiogenesis, which has a beneficial effect on reducing peripheral vascular resistance, asymptomatic HTLV-1 carrier status could be inversely associated with hypertension only among those with high platelet counts [19] (Figure 3f,g). Furthermore, angiogenesis is necessary for the development of structural atherosclerosis [4].

Because hypertension could be prevented by activating endothelial repair, among asymptomatic HTLV-1 carriers without hypertension, platelet count could be positively associated with structural atherosclerosis but in not those without HTLV-1 infection [19] (Figure 3b,c). Furthermore, among non-hypertensive individuals, HTLV-1 infection is significantly positively associated with structural atherosclerosis in those with high platelet counts but not in those with low platelet counts (Figure 3d,e). These associations indicate that the progression of structural atherosclerosis could prevent hypertension by activating endothelial repair.

In addition, in our previous study, genetic characteristics related to lower angiogenesis activity were revealed to be associated with a lower chance of establishing HTLV-1 infection [60]. Therefore, individuals with HTLV-1 infection might have higher angiogenesis activity than individuals without HTLV-1 infection, which prevents hypertension.

### 4.7. Potential Biological Reactions to Hypoxia and Oxidative Stress

Figure 4 shows the potential biological reactions to hypoxia and oxidative stress by focusing on reticulocytes, which act as an antioxidant. Adjustment for age-related physical changes account for biological reactions to hypoxia and oxidative stress because both hypoxia and oxidative stress are increased with the process of aging [45,46,61].

There are mainly two types of biological mechanisms that are responsible for age-related physical changes: mechanisms intended to compensate for decreased blood flow (oxygen supply) and mechanisms that increase antioxidant production.

Details about these biological reactions are described elsewhere [12]. Hypertension and the maintenance of the microcirculation, including angiogenesis, aim to compensate for decreased blood flow. Hypertension increases the effectiveness of existing blood vessels, while angiogenesis increases blood flow by building new vessels.

According to the reaction rate, hypertension and antioxidant production can be classified as rapid reactions, while angiogenesis can be classified as a delayed reaction. Hypertension and antioxidant production can be observed when hypoxia and oxidative stress levels are increased. In addition, since the progression of structural atherosclerosis might indicate angiogenesis activity, antioxidants might have the beneficial effect of preventing the development of structural atherosclerosis. However, both hypertension and the development of structural atherosclerosis are common reactions to hypoxia and oxidative stress. Hypertension could be positively associated with structural atherosclerosis.

Since reticulocyte count could act as a marker of antioxidant production, a cross-sectional study with data on reticulocyte count was conducted. In this study [34], reticulocyte count was revealed to be positively associated with hypertension (Figure 4a) and inversely associated with structural atherosclerosis (Figure 4c). This study also found a significant positive association between hypertension and structural atherosclerosis (Figure 4b). Since reticulocytes are a source of hemoglobin, which contributes to the oxygen supply directly, a significant positive association between hemoglobin and hypertension was also observed [35].

### 4.8. Hemoglobin and Hypertension by Platelet Levels (Figure 5)

Platelet levels indicate the level of endothelial repair activity [32]. Hemoglobin is positively associated with hypertension, which could indicate a rapid reaction to age-related physical changes [34,35].

Since efficient endothelial repair helps prevent hypertension [6,12,19], the positive association between hemoglobin levels and hypertension could be observed in individuals with low platelet counts and low or insufficient endothelial repair activity. A cross-sectional study with 222 Japanese men aged 60–69 years showed a positive association between hemoglobin levels and hypertension only in participants with lower platelet counts [36] (Figure 5a,c).

In addition, the absence of a rapid reaction to physical changes might indicate the presence of appropriate endothelial maintenance or repair. Among individuals with appropriate endothelial maintenance, the influence of a reduction in circulating CD34-positive cell count due to consumption should be limited [12,31,32]. Furthermore, since the proliferation of CD34-positive cells and platelets are activated upon endothelial injury [10,11,41], participants with rapid reactions (high hemoglobin levels) who cannot increase platelet levels efficiently (low platelet count) should have a shortage of CD34-positive cells (low CD34-positive cell count). Among such participants, low platelet counts might indicate insufficient endothelial repair, resulting in no significant correlations between hemoglobin levels and circulating CD34-positive cell counts (Figure 5d).

In a cross-sectional study with Japanese men, a significant positive correlation between hemoglobin levels and circulating CD34-positive cell counts was only observed among men with higher platelet counts [36] (Figure 5b,d). Therefore, non-hypertensive participants with high platelet counts might have appropriate endothelial repair that prevents hypertension.

### 4.9. BRAP rs3782886 and Platelet Count in Relation to Hypertension

Being an allele G carrier of SNP rs3782886 in BRAP is reported to be associated with myocardial infarction [62]. BRAP activates an inflammatory cascade through the activation of the NF-κB pathway and increases the risk of carotid atherosclerosis [63,64]. Platelet activation proteins could also be promoted via the activation of the NF-κB pathway [18]. Since platelet activation contributes to endothelial repair [32,40,41], the positive association between high platelet count and allele G of rs3782886 (Figure 6b), which was shown our study [37], indicates that allele G carriers have activated endothelial repair.

Furthermore, a high platelet count was positively associated with hypertension (Figure 6a), while there were no significant associations between allele G of rs3782886 and hypertension (Figure 6c) [37]. Therefore, for individuals who are allele G carriers of rs3782886 who might be at risk for structural atherosclerosis [63,64], activating endothelial repair might prevent hypertension.

### 4.10. BRAP rs3782886, ALDH2 rs671, Platelets, and Hypertension

Figure 7 shows the potential mechanism underlying vascular remodeling among BRAP rs3782886 and ALDH2 rs671. BRAP increases the risk of structural atherosclerosis by activating inflammation via the activation of the NF-κB pathway [63,64]. Activation of the NF-κB pathway promotes the production of platelet activation proteins [18]. Platelet count could act as an indicator of endothelial activity [32]. Therefore, the significant positive association between the minor allele of BRAP rs3782886 and a high platelet count [37] indicates that BRAP rs3782886 contributes to the activation of endothelial repair, which leads to the development of structural atherosclerosis.

ALDH2 is a key enzyme in alcohol metabolism. ALDH2 gene polymorphisms are rare in Caucasians, Africans, and Southeast Asians but are common in East Asians [22,23]. Strong LD values between BRAP rs3782886 and ALDH2 rs671 have been reported [38] (Figure 7c). ALHD2 rs671 was reported to influence drinking status [65]. Both ALDH2 rs671 and BRAP rs3782886 were revealed to be associated with never drinker status [23] (Figure 7h). The number of minor alleles of ALDH2 rs671 and BRAP rs3782886 were revealed to be significantly positively associated with never drinker status.

Ethanol exposure dramatically inhibits NF-κB [20] and directly attenuates platelet activation [21]. Hematopoietic activity that declines with age is important to determine the ability to produce platelets. Reticulocyte count could act as an indicator of hematopoietic activity. Therefore, among participants without decreased hematopoietic activity, platelet counts are significantly higher in never drinkers than in non-never drinkers [23] (Figure 7g).

Since BRAP rs3782886 influences endothelial repair activity, which is related to platelet count [37] and NF-κB pathway activity [63,64], the strong LD between BRAP rs3782886 and ALHD2 rs671 might influence endothelial repair activity.

The minor allele of BRAP rs3782886 and ALHD2 rs671 were revealed to be positively associated with platelet count only in older individuals with high reticulocyte counts [23] (Figure 7d). Reticulocyte count could influence the ability to produce platelets. BRAP rs3782886 and ALHD2 rs671 do not influence hematopoietic activity itself. Thus, no significant associations between the minor alleles of those SNPs and reticulocyte count were observed [23] (Figure 7f).

As a rapid reaction to adjust to increased oxidative stress, reticulocyte count increases, and hypertension occurs, leading to a significant positive association between reticulocyte count and hypertension [23] (Figure 7a). As a delayed reaction, endothelial repair is activated via increased platelet count [23] (Figure 7b).

Among individuals with activated endothelial repair, angiogenesis, which reduces peripheral blood pressure, also develops. Since the positive association between the minor alleles of those SNPs (rr37828886 and rs671) and platelet count were only observed in older individuals with high reticulocyte counts [23] (Figure 7d), the beneficial influence of hypertension prevention was observed in those with high reticulocyte counts [23] (Figure 7e).

Therefore, to enhance the beneficial influence of BRAP rs3782886 on hypertension prevention, the presence of ethanol is a hindrance. The strong LD between BRAP rs3782886 and ALHD2 rs671 could improve the efficiency of hypertension prevention dramatically when the influence of ethanol is avoided.

### 4.11. Structural Atherosclerosis, Functional Atherosclerosis, and LDL Cholesterol

Even when levels are within the normal range [66], by activating inflammation [67], LDLc directly contributes to the development of structural atherosclerosis [68]. Sufficient numbers of CD34-positive cells are mandatory for the development of structural atherosclerosis [44].

A shortage of CD34-positive cells leads to the development of functional atherosclerosis but not structural atherosclerosis [12,31,49]. LDLc was also reported to increase the proliferation of CD34-positive cells [69].

Therefore, LDLc could have a beneficial influence on preventing functional atherosclerosis that is related to CD34-positive cell shortages. In our cross-sectional study, LDLc levels were significantly positively associated with structural atherosclerosis and significantly inversely associated with functional atherosclerosis [39]. This study indicated that the progression of structural atherosclerosis is not always unfavorable to vascular health. The progression of structural atherosclerosis could have a partly beneficial effect on the maintenance of endothelial function.

Hypertension is a major risk factor for stroke in the Japanese population [70]. An autopsy study clarified that Japanese individuals living in Honolulu have significantly more atherosclerosis in the circle of Willis but less intra-parenchymal artery sclerosis and fewer cerebral infarctions than those living in Japan [71]. Therefore, in Japanese individuals, the progression of structural atherosclerosis indicates the development of angiogenesis, which might have a beneficial influence on hypertension prevention by reducing peripheral blood pressure.

With the beginning of an agricultural culture, ALDH2 rs671 has spread in East Asia [72]. The switch from a hunting and gathering culture to an agricultural culture changed eating habits dramatically. This change might have reduced serum LDLc levels. Since low serum LDLc levels might increase the risk of hypertension by reducing angiogenesis activity, BRAP rs3782886, which has a strong LD with ALDH2 rs671 [38], might have a beneficial influence on reducing the risk of hypertension by activating platelets. This may be the reason why agricultural populations acquired genetic characteristics that make them susceptible to alcohol.

### 4.12. Strengths of the Present Study

The present study focusing on circulating CD34-positive cells, platelets, HTLV-1, and SNPs in VEGF, BRAP, and ALDH2 has shown that active endothelial repair, which leads to the progression of structural atherosclerosis, partly indicates the prevention of hypertension.

Therefore, inhibiting the progression of structural atherosclerosis is not always a beneficial strategy for cardiovascular disease prevention. Unlike general epidemiological studies that have evaluated the harmful or preventive effects of atherosclerosis and hypertension, our epidemiological studies included a multifaceted analysis that can explain the harmful and preventive effects of structural atherosclerosis in a single targeted population.

Those multifaceted analyses clarified a potential novel mechanism underlying vascular remodeling (endothelial repair), which could explain the beneficial effect of structural atherosclerosis.

## 5. Perspectives

Oxidative stress, which induces both hypertension [7] and structural atherosclerosis [8], increases with aging [45,46,61]. Therefore, the need for endothelial repair increases with aging. Circulating CD34-positive cells, which play a major role in endothelial repair [12], are necessary for the development of structural atherosclerosis [44]. However, aging is also associated with declines in hematopoietic activity that result in a reduced ability to produce CD34-positive cells [47,48]. Thus, in an aged society, there is likely higher cardiovascular risk related to a shortage of circulating CD34-positive cells.

Since aggressive endothelial repair is associated with the development of both structural and functional atherosclerosis while insufficient endothelial repair furthers functional atherosclerosis but not structural atherosclerosis [12,31,49], functional atherosclerosis without structural atherosclerosis could have a great impact on cardiovascular disease in an aged society.

Structural atherosclerosis and functional atherosclerosis are generally taken to be essentially the same clinical condition [73]. However, from the view of endothelial repair activity, there is an important difference. However, this concept has not become widespread yet. Evaluating vascular health using a combination of structural atherosclerosis and functional atherosclerosis could be efficient in daily clinical practice [74,75]. Further investigation based on this concept is necessary.

## 6. Limitations

Height [49,76] and height loss [77] could be associated with the capacity to produce circulating CD34-positive cells. Height and height loss are associated with hypertension [78]. Height was also found to be inversely associated with structural atherosclerosis for overweight but not for non-overweight men [79]. However, our present study did not evaluate the influence of height loss on evaluating the activity of hypertension prevention related endothelial repair. In addition, thyroid cysts are associated with height, structural atherosclerosis, and hypertension [80,81,82,83]. Therefore, thyroid cysts can also have an effect on hypertension and structural atherosclerosis. The influence of height loss and thyroid cysts on the beneficial influence of hypertension prevention related to the development of structural atherosclerosis is unknown.

## 7. Conclusions

The progression of structural atherosclerosis could partly indicate angiogenesis activity. Since angiogenesis reduces peripheral blood pressure, the progression of structural atherosclerosis contributes to the prevention of hypertension. However, a positive association between structural atherosclerosis and hypertension has been observed. Increased oxidative stress is a common risk factor for the progression of structural atherosclerosis and hypertension. Thus, there is a novel mechanism underlying vascular remodeling. This novel mechanism could be clarified by focusing on endothelial repair activity. Further investigation focusing on endothelial repair activity is necessary.

## Data Availability

We cannot publicly provide individual data due to participant privacy, according to ethical guidelines in Japan. Additionally, the informed consent that was obtained does not include a provision for publicly sharing data. Qualifying researchers may apply to access a minimal dataset by contacting the office of data management at ritouken@vc.fctv-net.jp. Information for data requests is also available at http://www.med.nagasaki-u.ac.jp/cm/ (accessed on 23 May 2023).
